# Draft Genome Sequence of the Bioelectricity-Generating and Dye-Decolorizing Bacterium *Proteus hauseri* Strain ZMd44

**DOI:** 10.1128/genomeA.00992-13

**Published:** 2014-01-16

**Authors:** Nan Wang, I-Son Ng, Po Ting Chen, Yuzhe Li, Yi-Chung Chen, Bor-Yann Chen, Yinghua Lu

**Affiliations:** Department of Chemical and Biochemical Engineering, College of Chemistry and Chemical Engineering, Xiamen University, Xiamen, Chinaa; Key Laboratory for Chemical Biology of Fujian Province, Xiamen University, Xiamen, Chinab; Department of Biotechnology, Southern Taiwan University of Science and Technology, Yungkang, Tainan City, Taiwanc; Department of Chemical and Materials Engineering, National I-Lan University, I-Lan, Taiwand

## Abstract

*Proteus hauseri* ZMd44 (CGMCC 6746), as a crucial biodecolorizing, bioelectricity-generating, and copper-resistant bacterium, is distinguished from the urinary pathogens *Proteus penneri* and *Proteus mirabilis*. To further investigate the genetic functions of this strain, the genome sequence and annotation of its open reading frames, which consist of 3,875,927 bp (G+C content, 38.12%), are presented here.

## GENOME ANNOUNCEMENT

*Proteus hauseri* ZMd44 is a new classification of the *Proteus* genus (1), which was found in Lanyang Plain, northeast Taiwan. It has been used for application in microbe fuel cells (MFCs) with wastewater using azo dye Reactive Blue 160 (RBu160) (2). Moreover, the interactive characteristics of bioelectricity generation and dye decolorization in MFCs have been established (3). Recently, *P. hauseri* ZMd44 has been found to express McoA-laccase by induction with copper sulfate (4). Moreover, *P. hauseri* cells were significantly suppressed and swarming motility was inhibited with supplementation of Cu(II). This implies that its copper-related genes potentially contain vital therapeutic functions, since *P. hauseri* lost capabilities to cause swarming motility and urinary tract infections in the presence of Cu(II). This is why the genome sequencing for this new species is of great importance and significance.

High-throughput DNA sequencing of *P. hasueri* ZMd44 was conducted on the Illumina GA IIx 90-bp paired-end platform, with an average insert size of 250 bp, at Yourgene Bioscience (Taiwan). In total, 14,455,522 reads were achieved, resulting in 3.73-fold genomic coverage. The reads were filtered to remove adapter sequences and achieve the quality trim of at least 35 bp. A draft genome was generated by *de novo* assembly, using a high-volume-read-accommodating algorithm by storing data in de Bruijn graphs (5). This genome is a single circular chromosome of 3,875,927 bp with a mean G+C content of 38.12%. Fifty-one contigs with an N_50_ value of 197,041 and a largest contig of 1,426,865 bp were constructed. Gene prediction using GeneMark (6) revealed a total of 3,147 open reading frames (ORFs). The predicted proteins were annotated by implementing a BLASTp search against the NCBI NR database.

A comparative genome analysis of *P. hauseri* ZMd44 with the closely related *Proteus mirabilis* HI4320 (7) revealed that the strains are similar, with 2,274 known protein-coding genes, 490 hypothetical protein-coding genes, and 383 no-hits genes. The known coding genes for proteins and enzymes are involved in the three catalogues of biological process (44.9%), cellular component (23.5%), and molecular function (31.6%). On the other hand, only four ORFs encode proteins that are predicted to be involved in the copper resistance properties of *P. hauseri* ZMd44. The proteins are copper homeostasis protein CutC, copper resistance protein Crp, multicopper oxidase Mco, and repressor protein FtsI. Among these, the highest sequence identity of Crp to other pathogens microbes is only 33.3% with that of the PcoD from *Escherichia coli* strain K-12 substrain W3110 (accession no. YP_490102), followed by 12.8% with that of CopD from *Shewanella putrefaciens* CN-32 (accession no. YP_001181786). Thus, the lower similarity of the copper-resistant protein has suggested that Crp is the key protein of *P. hauseri* ZMd44 for copper resistance. This draft genome sequence helps accelerate the understanding of the characteristics of this strain not only in application of MFC and bioremediation processes, but also by potentially using the copper-related genes in therapeutic therapy of urinary tract infection caused by *Proteus*  species.

### Nucleotide sequence accession number.

The whole-genome shotgun project for *P. hauseri* ZMd44 has been deposited in GenBank under the accession no. AWXP00000000.
